# Microchemical provenancing of prey remains in cormorant pellets reveals the use of diverse foraging grounds

**DOI:** 10.1002/jwmg.22248

**Published:** 2022-05-08

**Authors:** Johannes Oehm, Andreas Zitek, Bettina Thalinger, Anastassiya Tchaikovsky, Johanna Irrgeher, Thomas Prohaska, Michael Traugott

**Affiliations:** ^1^ Department of Zoology University of Innsbruck Technikerstraße 25 6020 Innsbruck Austria; ^2^ FFoQSI GmbH—Austrian Competence Centre for Feed and Food Quality, Safety and Innovation Technopark 1D 3430 Tulln Austria; ^3^ Center of Biodiversity Genomics University of Guelph 50 Stone Road East Guelph N1G2W1 Canada; ^4^ Department of Analytical Chemistry University of Natural Resources and Life Sciences Muthgasse 18 1190 Vienna Austria; ^5^ Department of General Analytical and Physical Chemistry, Montanuniversität Leoben Franz‐Josef‐Straße 18 8700 Leoben Austria; ^6^ Chair of General and Analytical Chemistry, Montanuniversität Leoben Franz‐Josef‐Straße 18 8700 Leoben Austria

**Keywords:** Alpine Foreland, otolith chemistry, *Phalacrocorax carbo sinensis*, piscivores, prey, provenance

## Abstract

Piscivorous birds in aquatic ecosystems exert predation pressure on fish populations. But the site‐specific impact on fish populations, including stocked and commercially used fish species, remains disputed. One of the key questions for the management of piscivorous birds and fish is determining the origin of prey and thus which fish populations are targeted by the birds. We addressed this question by provenancing otoliths (earstones) of fish obtained from regurgitated pellets of piscivorous birds by otolith microchemistry analysis. We retrieved otoliths from regurgitated pellets of great cormorants (*Phalacrocorax carbo sinensis*) collected every 2 weeks for 2 years from breeding and roosting colonies at Chiemsee in Bavaria, Germany, and classified them according to family or species. We collected water samples from Chiemsee and potential surrounding foraging grounds. We measured the strontium (Sr) ^87^Sr/^86^Sr isotope ratio and Sr mass fraction of water and otoliths using (laser ablation) inductively coupled plasma‐mass spectrometry. We assigned otoliths from regurgitated pellets to habitat clusters of origin by comparing the Sr isotopic and elemental composition of otoliths and waterbodies. In 36% of cormorant pellets collected at Chiemsee, prey was assigned to waterbodies distinct from Chiemsee. Furthermore, cormorants used different foraging sites during 1 day. Microchemical provenancing of prey remains can contribute to identifying foraging sites of piscivorous birds and to what extend the birds switched among foraging sites.

## Graphical abstract

Microchemical provenancing was successfully applied to otoliths exposed to digestion by piscivorous birds and it was possible to clarify on a small scale from which waterbodies or habitat clusters the birds obtain their food. By the use of prey provenancing, site‐specific management plans can be developed and the effect of management measures on piscivores, fish, and their ecosystems can be predicted.

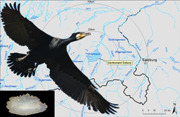

For the conservation and management of animals, it is necessary to understand their functional role and connectivity within an ecosystem. Trophic interactions are a driver of the distribution and abundance of animal species with the establishment and stability of populations depending, among other things, on food acquisition and losses to predators (de Ruiter and Moore 2005). To evaluate predator‐prey interactions, researchers must identify the prey species taken, the amount consumed, where the prey was caught, and how prey populations are affected. This can be ambiguous for highly mobile predators such as seabirds, marine mammals, or bats when foraging takes place at secluded or hidden locations and direct observation is impossible (Carss 1997, Clare et al. 2011, Torres 2013). Provenancing of prey remains allows determination of the predator's foraging habitats, its targeted prey population, and the predator's foraging range. Locating prey populations used by predators is vital to assess and predict their impact on the prey population and to develop management plans for prey and predators (Glen and Dickman 2005, Tang et al. 2009, Russell et al. 2012, Jarillo et al. 2020). A variety of approaches have been used to provenance animal and human food sources (Kelly et al. 2005, Gopi et al. 2019, Krajnc et al. 2021), including the analysis of elements, stable isotopes and fatty acids, and profiling DNA.

The application of these different approaches on prey remains poses specific challenges. For example, soft tissues such as muscle and fat can be fully digested in dietary samples and, consequently, are not available for fatty acid profiling. Furthermore, semi‐digested and therefore fragmented DNA might not provide an adequate quality to identify potential genetic variations in remains of ingested prey (King et al. 2008). On the other hand, indigestible prey remains can be used for trace element and stable isotope analysis to determine the origin of the prey. Stable isotope analysis of carbon (C) and nitrogen (N) has traditionally been used to determine trophic relationships and spatial distributions of animals (Gladyshev 2009, Boecklen et al. 2011, Traugott et al. 2013, Quinby et al. 2020). Regarding prey provenancing, Seco et al. (2016) used δ^13^C signatures of short‐finned squid (*Illex argentinus*) beaks retrieved from regurgitates of seabirds to assess if the prey originated from the south Atlantic or Antarctic waters. The differences in δ^13^C values between south Atlantic and Antarctic waters reflected in squid beaks were significant, which allowed provenancing of the ingested squid to one of these oceans. Bugajski et al. (2013) reported C and N isotopes could discriminate among perch (*Perca* spp.) populations of 5 big Canadian lakes to successfully provenance fish regurgitated by double‐crested cormorants (*Phalacrocorax auritus*). The accuracy for provenancing prey on a smaller spatial scale using C and N isotopes is often limited, however, because there is usually little variation in the isotopic signatures between respective geographic regions (Turchini et al. 2009, Carter et al. 2015).

Bearhop et al. (1999) suggested applying multiple isotope ratios to identify the origin of fish prey in different freshwater ecosystems. In particular, strontium (Sr) isotope ratios (expressed as the amount of the isotope ^87^Sr divided by the amount of the isotope ^86^Sr in a sample; ^87^Sr/^86^Sr) represent a useful marker for identifying the origin of fish remains (Campana 1999). This index relies on waterbodies exhibiting different Sr isotopic composition in their geological bedrock (Faure and Mensing 2005). These differences are reflected in calcified structures of fish (Campana 1999). Strontium isotope ratios in fish otoliths (earstones) allowed successful tracing of fish back to their natal freshwater origin (Tomas et al. 2005, Barnett‐Johnson et al. 2008, Zitek et al. 2010, Avigliano et al. 2020). It has not yet been clarified, however, how the microchemistry of calcified structures is affected by the digestion of predators. Phelps et al. (2009) and Kemp et al. (2011) reported microchemistry and isotope ratios in otoliths were persistent when digested by fish and seals, respectively, but this has not been tested on otoliths digested by seabirds.

Seabirds remove considerable fish biomass from aquatic ecosystems, which often results in conflicts with fisheries (Sydeman et al. 2017). In the northern hemisphere the most disputed species among piscivorous birds is the great cormorant (*Phalacrocorax carbo sinensis*; cormorant). Cormorants occur across Central European freshwater sources throughout the year (van Eerden et al. 2012), potentially move among different freshwater foraging sites (Bugajski et al. 2013), and can cover distances up to 70 km/day when foraging among inland waterbodies (Suter 1993). This makes them one of the most prominent piscivores in the Alpine Foreland. The Alpine Foreland is characterized by a high number of stagnant and running waters and catchments on a highly diverse geological background. In the Bavarian Foreland of the Alps, Zitek et al. (2021) classified water samples of 19 different waterbodies (rivers and lakes) within a 50‐km range to 7 clusters based on ^87^Sr/^86^Sr isotope ratios and Sr/Ca ratios. The differences among analyzed waters were also reflected in otoliths of 16 fish species typically inhabiting these waterbodies, indicating that the prerequisite for small‐scale microchemical provenancing of fish hard parts are met in this region. We employed microchemical provenancing to fish otoliths obtained from regurgitated pellets of cormorants (i.e., pellets). Cormorants regurgitate these pellets daily to excrete indigestible prey remains such as bones and otoliths (Zijlstra and van Eerden 1995). Our objective was to identify the birds' foraging grounds by comparing the isotope composition of otoliths from cormorant pellets to those found in local waterbodies. We predicted that pellets containing lacustrine species are indicative of foraging at Chiemsee and assessed to what extent cormorants use foraging habitats outside of Chiemsee.

## STUDY AREA

The study area is located in Chiemgau, which is a cultural landscape in southern Germany in the Alpine Foreland. Chiemgau is bordered by the river Inn to the west, the river Traun to the east, and the border with Austria to the south. The northern boundary is set where the loess‐covered alto moraine of the hill country between the rivers Alz an Inn meets the top moraine of the Chiemgau. Chiemgau is mainly hilly with more mountainous areas in the south and the elevation ranges from 500 m to 1,800 m (Bayerisches Landesamt für Umwelt 2011). The region is characterized by temperate continental climate with the 4 distinct seasons: spring (Mar–Jun), summer (Jun–Sep), autumn (Sep–Dec), and winter (Dec–Mar). The average annual temperature in the Bavarian Alpine Foreland is 8.2°C with average temperatures of −0.3°C in winter and 16.6°C in summer and the annual precipitation in the study region is on average 999 mm/year (Bayerisches Landesamt für Umwelt 2021).

Forested landscapes of the lowland are dominated by deciduous forests (beech [*Fagus sylvatica*], oak [*Quercus* spp.], sycamore maple [*Acer pseudoplatanus*], ash [*Fraxinus excelsior*]), which mix and change to coniferous forests (spruce [*Picea abies*], fir [*Abies alba*], pine [*Pinus sylvestris*], larch [*Larix decidua*]) in higher altitudes. The vegetation along the shoreline of lakes and rivers is composed of lowland tree species with isolated areas of primary riparian forest (willow [*Salix* spp.], alder [*Alnus* spp.], poplar [*Populus* spp.]) and common reed (*Phragmites australis*) beds in shallow littoral zones. Agricultural lands are mainly meadows and fields for the production of hay and corn.

The study area has a large wildlife diversity, with roe deer (*Capreolus capreolus*), red deer (*Cervus elaphus*), wild boar (*Sus scrofa*), hare (*Lepus europaeus*), red fox (*Vulpes vulpes*), and European badger (*Meles meles*) being the dominant mammalian species in forests and agricultural lands. Crows (carrion crow [*Corvus corone*], common raven [*Corvus corax*]), tits (great tit [*Parus major*], blue tit [*Cyanistes caeruleus*]), warblers (chiffchaff [*Phylloscopus collybita*], blackcap [*Sylvia atricapilla*]), thrushes (blackbird [*Turdus merula*], robin [*Erithacus rubecula*]), and finches (chaffinch [*Fringilla coelebs*], greenfinch [*Chloris chloris*]) are common bird species in the forests. The most common waterbird species in this region are gray goose (*Anser anser*), ducks (mallard [*Anas platyrhynchos*], tufted duck [*Aythya fuligula*], common pochard [*Aythya farina*]), gulls (black‐headed gull [*Larus ridibundus*], common gull [*Larus canus*], yellow‐legged gull [*Larus michahellis*]), herons (gray heron [*Ardea cinerea*], great white egret [*Egretta alba*]), rails (coot [*Fulica atra*], gallinule [*Gallinula chloropus*]), goosander (*Mergus merganser*), great crested grebe (*Podiceps cristatus*) and the great cormorant. Chiemsee provides habitat for a wide range of waterbirds and is therefore listed as a wetland of international importance according to the Ramsar Convention (Convention on Wetlands of International Importance especially as waterfowl habitat). Lakes in the study region are mainly inhabited by cyprinids (roach [*Rutilus rutilus*], rudd [*Scardinius erythrophthalmus*], bream [*Abramis brama*], carp [*Cyprinus carpio*]), perch (*Perca fluviatilis*), and whitefish (*Coregonus* spp.), while rivers are dominated by salmonids (brown trout [*Salmo trutta*], rainbow trout [*Oncorhynchus mykiss*], grayling [*Thymallus thymallus*]).

Chiemsee has a water expanse of 79.9 km² and is the largest lake in southern Germany at an elevation of 518 m. The lake is used for watersports, commercial and recreational fisheries, and tourist excursions and is an important breeding and roosting site for cormorants and other waterbirds. Hence, cormorants occur at the lake throughout the year and are regularly present in surrounding stagnant and running waterbodies. In previous studies on the prey choice of cormorants at Chiemsee, mainly hard part remains of typical lake species were found in pellets (Keller 1995, 1998; Oehm et al. 2017), indicating that these birds mainly use this lake as their foraging site.

**Figure 1:**
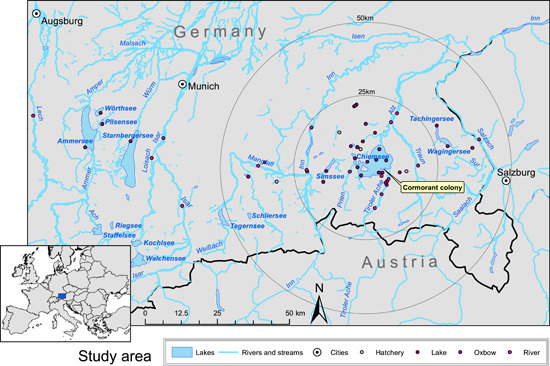
The study area in southern Bavaria with lakes, rivers, and the location of the cormorant breeding colony at Chiemsee, 2012–2014. The lower left inset shows the location of the study area within Germany. Water sampling sites are indicated as filled circles.

During the investigations of this study between March 2012 and February 2014, the breeding colony at Chiemsee consisted of 2 sub‐colonies (N 47.862839, E 12.503541 and N 47.859971, E 12.509115; Figure 1) including ≤111 breeding pairs (Geiersberger 2013), both of them located in the estuary of the river Tiroler Achen, the main inflow of the lake. There are several roosting sites during autumn and winter and the most regularly used sites are located at Krautinsel (N 47.869639, E 12.418735), a nearby gravel bank (N 47.863077, O 12.419983) and at the breeding colony (Figure 1). The estuary of the river Tiroler Achen is embedded in the nature sanctuary Achen‐Delta. Permission to enter this sanctuary for fieldwork was provided by the nature conservation authority of the district government of Upper Bavaria.

## METHODS

### Water samples and cormorant pellets

We collected water samples from 53 sites in 41 different waterbodies, which represent the main lentic and lotic waterbodies in a 50‐km radius around the cormorant colony at Chiemsee (Figure 1). We collected additional samples in a 50‐km radius around Ammersee because this lake hosts another cormorant breeding and roosting colony and birds might move from the area around Ammersee to Chiemsee. We sampled most of the waterbodies at a single location, while Alz, Inn, Isar, and Tiroler Ache rivers were sampled in multiple locations (Figure 1; Table S1 available in Supporting Information). We sampled Chiemsee at 7 different locations (Figure 1; Table S1). At each sampling site, we took 3 100‐ml water samples in acid‐washed polyethylene bottles (cleaned with nitric acid and rinsed with deionized water) using a hand‐dipping device where the open bottles could be mounted. We collected water samples from Chiemsee at Chieming, Felden, Prien, and Seebruck at 1 m and 12 m using a Schindler‐Patalas water sampler (Uwitec, Tiefgraben, Austria) from which 3 subsamples were bottled. Altogether, we took 90 water samples, kept them in a cooler during transport, and stored them in the laboratory at −28°C until measurement.

We collected cormorant pellets every 2 weeks from March 2012 to February 2014 at 4 cormorant breeding and roosting locations at Chiemsee (Figure 1). We collected 4,894 pellets during 42 sampling dates. We considered the collected pellets were produced by cormorants because we did not observe other pellet‐producing species roosting or breeding together with the cormorants. We packed each pellet in a small plastic bag, stored them in a cooler, and transferred them to the laboratory at the University of Innsbruck for storage at −28°C. For proof of principle and to test whether the provenancing of otoliths is affected by the birds' digestion, we fed whitefish caught in Chiemsee to captive cormorants during a feeding experiment in the zoological botanical garden Wilhelma in Stuttgart, Germany (Thalinger et al. 2017). We fed 30 whitefish to 4 cormorants in 3 meals (i.e., each bird fed on 3–4 whitefish/meal). We collected the birds' pellets (*n* = 28) the next morning and stored them the same way described above.

### Prey identification and sample selection

We defrosted the pellets from the field and from the feeding experiment and transferred each into a separate 50‐ml Greiner tube. Depending on the pellet's size, we added 3–8 ml of buffer solution (TES buffer and Proteinase K [20 mg/ml] in a ratio of 190:1). Afterwards we vortexed the samples and put them into an incubator at 58°C for 24 hours.

For morphological hard part analysis, we sieved and rinsed the dissolved pellet with distilled water and sorted out identifiable hard parts such as otoliths, pharyngeal teeth, chewing pads, jaws, scales and pre‐opercula for prey identification. We identified the obtained key bones to the highest taxonomic level possible using the identification keys of Härkönen (1986), Veldkamp (1995), Knollseisen (1996), and Cech (2006) and by using fish bone reference collections (W. Suter, Swiss Federal Research Institute, Birmensdorf, Switzerland; J. Trauttmansdorff, Otto‐König Institute, Stockerau, Austria; Bavarian State Collection of Zoology, Munich, Germany). We classified identified fish into species that typically inhabit Chiemsee and stagnant waters of the study region or that predominately inhabit running waters.

**Figure 2:**
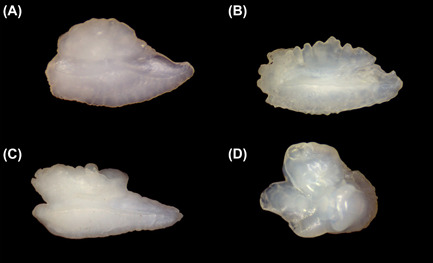
Otoliths of salmonids (A), perch (B), whitefish (C), and cyprinids (D) caught in the study region around the cormorant colony at Chiemsee in Germany, 2013–2014.

We chose 3 pellets from the feeding experiment containing intact otoliths of the fed whitefish for microchemical measurement. From the samples collected at Chiemsee, we selected up to 16 pellets/sampling date as far as this number could be obtained at the respective sampling date. In a first step, we chose every pellet containing remains of riverine fish species that usually do not occur in Chiemsee such as brown trout, grayling, rainbow trout, brook charr (*Salvelinus fontinalis)*, barbel (*Barbus barbus*), nase (*Chondostroma nasus*), ruffe (*Gymnocephalus* spp.), minnow (*Phoxinus phoxinus*), bullhead (*Cottus gobio*), dace (*Leucicus leuciscus*), and chub (*Squalius cephalus*). We selected the remaining number of pellets (16/date) from pellets containing typical species of Chiemsee such as whitefish, perch, and roach. Altogether, we examined 597 of the field‐collected pellets for the presence of intact sagittae (i.e., the largest of the 3 pairs of otoliths) of salmonids, whitefish, perch, and the lapilli of cyprinids (Figure 2). Per pellet, we selected 1 otolith of the respective fish taxon, which we sonicated for 5 minutes, and air dried overnight.

For the microchemical analysis, we grouped otoliths regarding priority of interest: group 1 included trout and grayling (i.e., salmonids), which are mainly inhabits of riverine ecosystems and were thus indicative of foraging in rivers and streams. For each pellet containing an otolith of trout or grayling, we measured a second otolith belonging to a different taxon if available. This was the case in 30 pellets and enabled us to test whether both fish were caught in the same waterbody and, in case the salmonid otolith gave an indefinable signal, to test if both fish species were taken from the same water. In group 2 we chose whitefish because they occur in only 7 lakes in a 50‐km range around the cormorant colonies at Chiemsee, which limits the number of possible foraging grounds for this species. In contrast, perch (group 3) is a common ubiquitous species in lakes, ponds, and rivers of the study region. We chose their otoliths because perch was abundant in analyzed cormorant pellets. Another frequently consumed prey taxon is the family of cyprinids (group 4). The lapilli of cyprinids cannot be identified on a species‐specific level; therefore, they were the least favorable option for measurement. In 16 cases no intact otoliths of the before mentioned species were present in the selected pellets; therefore, we chose the present otoliths of burbot (*Lota lota*), eel (*Anguilla anguilla*), pike (*Esox lucius*), pikeperch (*Sander lucioperca*), or ruffe for microchemical analysis.

Following this selection procedure, we chose 631 otoliths consisting of 3 whitefish otoliths from the feeding experiment and 628 from the field for microchemical analysis. These 628 otoliths consisted of otoliths of perch (*n* = 267), whitefish (*n* = 189), cyprinids (*n* = 103), salmonids (*n* = 53), pike (*n* = 6), ruffe (*n* = 4), burbot (*n* = 3), pikeperch (*n* = 2), and eel (*n* = 1).

### Microelemental and Sr isotopic analysis

We transported all water samples frozen to the mass spectrometry facilities of the University of Natural Resource and Life Sciences Vienna, Tulln, Austria. We defrosted the samples, filtered them via a cellulose acetate filter membrane (Minisart 0.45‐µm syringe filter units, Minisart, Sartorius, Göttingen, Germany) and acidified them to obtain 2% nitric acid. We used the certified reference material SLRS5 river water (National Research Council, Ottawa, Canada) for method validation along with all sample preparation steps. We measured strontium (Sr) and calcium (Ca) in standard mode using a quadrupole inductively coupled plasma mass spectrometer ICP‐QMS (ELAN DRC‐e or NexION300D, Perkin Elmer, Waltham, MA, USA), and considered the elemental amount ratios of Sr/Ca ratio for further analyses.

Prior to Sr isotope ratio measurements, we performed a Sr/matrix separation using a Sr‐specific extraction resin (Triskem, Bruz, France; Horsky et al. 2016). We measured the Sr isotope ratios of the water samples using a multi‐collector inductively coupled plasma mass spectrometer (MC ICP‐MS, Nu Plasma HR, Nu Instruments, Wrexham, United Kingdom). We corrected the ^87^Sr/^86^Sr isotope ratios for instrumental isotopic fractionation using external intra‐elemental calibration using the certified reference material NIST SRM 987 in a standard‐sample bracketing mode (Irrgeher and Prohaska 2016). We determined expanded uncertainties (*U*, *k* = 2) corresponding to a coverage factor of 95% according to EUROCHEM guidelines (Ellison et al. 2012, Horsky et al. 2016).

We cleaned otoliths collected from cormorant pellets that we selected for microchemical analysis from adherent tissue in a petri dish filled with reagent grade I water (18,2 MΩ cm, TKA‐GenPure, Niederelbert, Germany), sonicated them for 8 minutes in an ultrasonic bath (model number UD80SH‐2L, Eumax Technology, Shenzhen, China), and air‐dried and stored them in pre‐cleaned polypropylene vials until further processing.

We transferred otoliths to individually labeled cut pieces of microscope slides (12 mm × 12 mm), fixed them with thermoplastic glue (Crystalbond^TM^ 509, Aremco Products, New York, NY, USA) and heated them on a heating plate (Type RCT basic, IKA Labortechnik, Staufen, Germany) with sulcus side up. After fixation, we flattened a small portion on the upper side of the otolith for the laser ablation using a 30‐µm and 3‐µm lapping film (3 M™ 266X PSA, St. Paul, MN, USA). As standard reference materials, we used fish otolith powder FEBS‐1 (National Research Council of Canada, Ottawa, Canada) and calcium carbonate MACS‐3 (U.S. Geological Survey, Reston, VA, USA), which were pressed into pellets.

We performed simultaneous multi‐element and isotope ratio measurement on the upper side of the otoliths for a length of 160 µm using laser ablation split stream ICP‐MS/MC ICP‐MS according to Prohaska et al. (2016). We applied spot sizes between 100 µm and 150 µm and a scan speed of 2 µm/second. We used the certified reference materials FEBS‐1 and MACS‐3 for elemental quantification, Sr isotope ratio calibration, and method evaluation and measured references at the beginning and end of the analysis. We performed data reduction for determining the ^87^Sr/^86^Sr isotope ratios and Sr mass fractions following the protocol described by Prohaska et al. (2016). All uncertainties correspond to the expanded uncertainty (*U*, *k* = 2).

### Statistical analysis

For displaying potential differences in ^87^Sr/^86^Sr isotope ratios in water samples, we ranked the values and plotted them together with expanded uncertainties (*U*, *k* = 2) to identify so‐called strontium isotope groups (SIGs) according to Brennan et al. (2015). In a next step, to inspect the distribution of ^87^Sr/^86^Sr isotope and Sr/Ca ratios of water samples, we used 2‐parameter plots. Subsequently, we used water chemistry data for building habitat clusters with similar characteristics using the Ward algorithm (IBM SPSS Statistics for Windows, version 24.0, Chicago, IL, USA) with values standardized between 0 and 1 to balance the different dimensions of the 2 parameters. We built water clusters containing the potential habitats for each taxon separately using existing information about their occurrence. Information on species occurrence per waterbody was provided by Kottelat and Freyhof (2007), Schmidt (2012), and fishermen. For cyprinids and salmonids, we did not differentiate habitat by species level. Following an approach suggested by Kern et al. (2017), we refined clusters based on analytical considerations. As a result, we added the final cluster membership to the waterbodies and visualized them as signatures in the 2‐parameter bi‐plots.

For discriminating the otoliths of unknown origin according to the habitat chemistry data, we used non‐parametric discriminant analysis using the DISCRIM procedure of SAS (SAS Institute, Cary, NC, USA): kernel = Epanechnikov, with the radius adapted to meet the data distribution; radius = 2 for pike, radius = 3 for cyprinids, burbot, trout, and grayling, radius = 4 for perch, radius = 5 for eel, radius = 7 for whitefish.

Following the validated assumption that the water ^87^Sr/^86^Sr isotope ratio and the Sr/Ca ratios are reflected in otolith chemistry according to their natural variation, we performed non‐parametric discriminant analysis using water data as training data and otolith data as test data to associate the otoliths of unknown origin to the water clusters. For this analysis, the water data and otolith data needed to be standardized between 0 and 1. We standardized Sr isotope data of all water samples and all otoliths (of unknown origin and from Zitek et al. [2021]) together between 0 and 1 considering that minimum and maximum values of water and otolith samples were overlapping within their associated expanded uncertainties. A different approach had to be used for Sr/Ca ratios because this ratio in water is due to discrimination effects only reflected in otoliths as a certain proportion (Campana 1999) and species‐specific differences have been documented by Zitek et al. (2021). First, we determined the minimum and maximum values of the Sr/Ca ratio in all habitats. Next, based on regression formulas provided by Zitek et al. (2021), we calculated the potential minimum and maximum Sr concentration values of perch, whitefish, cyprinids, pike, eel, and burbot and compared them to the values of the analyzed otoliths. To adjust the spread between water and otolith samples and to make them comparable, we used theoretical minimum and maximum Sr mass fractions expected in otoliths from these habitats for the standardization of otolith Sr mass fractions between 0 and 1 where needed. We used the following formulae from Zitek et al. (2021) to calculate the Sr mass fraction of otoliths (*y*) out of the Sr/Ca ratio in the water (*x*): *y* = 94,822*x* + 59.134 for perch and pike (Zitek et al. [2021]), *y* = 17,5913*x* − 30.384 for whitefish, *y* = 136,057*x* + 72.129 for cyprinids, and *y* = 16,8121*x* − 213.818 for burbot. To come up with an adequate range of data using the standardization procedure, we calculated a potential maximum of 950 µg/g Sr for pike otoliths, and introduced a potential minimum of 300 µg/g Sr and a potential maximum of 1,350 µg/g for burbot. For salmonids and eel no clear relation could be documented by Zitek et al. (2021) because these fish are often stocked. For these species, the existing values were standardized between 0 and 1 in direct relation to the water samples assuming that the fish and water samples had a similar span. For validating the classification of otoliths of unknown sources to the clusters, we included the otolith samples of known origin from Zitek et al. (2021) in the analysis with the associated habitat cluster numbers.

We calculated and plotted the share of pellets containing hard part remains of typical lacustrine or riverine prey species using Microsoft Excel (Microsoft Corporation, Redmond, WA, USA). We statistically compared differences in the assignment of otoliths between taxa and seasons with χ^2^ tests using IBM SPSS Statistics 26.

## RESULTS

**Figure 3:**
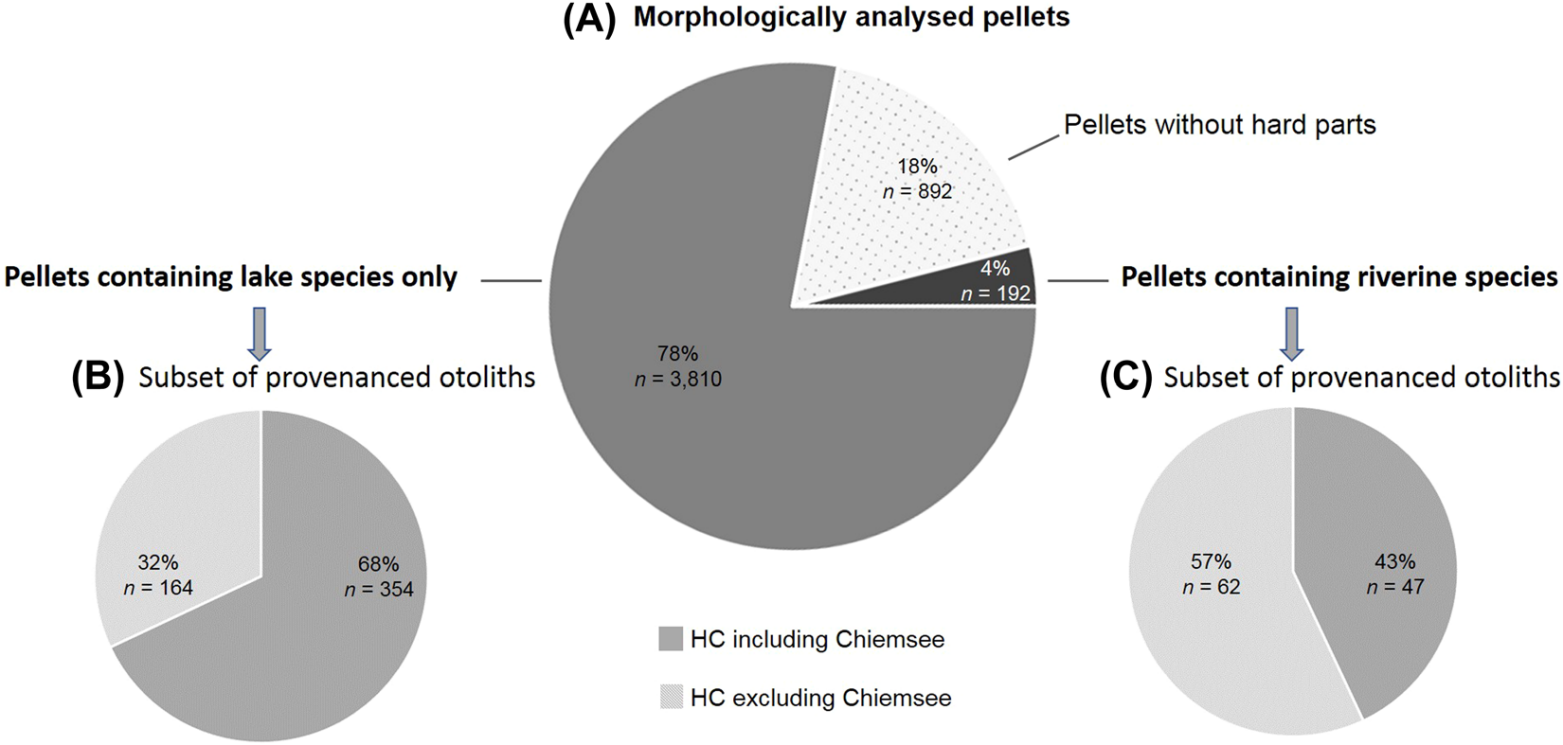
Share of cormorant pellets collected at Chiemsee in Germany, 2012–2014, containing hard part remains (A), remains of typical lake species only (B), or remains of riverine prey species (C), and assignment of a subset of these 2 groups to habitat cluster (HC) including and excluding Chiemsee based on microchemical analysis.

Out of the 4,894 field‐collected and morphologically analyzed pellets, 82% (*n* = 4,002) contained remains of ≥1 fish (Figure 3A). In 95% (*n* = 3,810) of these pellets the detected prey were typical lacustrine and ubiquitous fish species, whereas typical riverine species of the study region such as brown trout, rainbow trout, grayling, barbel, nase, ruffe, bullhead, minnow, dace, or chub were in 5% (*n* = 192; Figure 3A).

**Figure 4:**
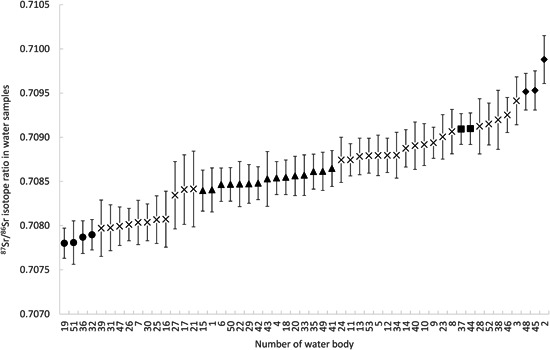
The 4 distinguishable strontium isotope groups (SIGs) after Brennan et al. (2015) as determined by non‐overlapping expanded uncertainties (*U*, *k* = 2) represented by different filled signatures; habitats with overlapping uncertainties that could not be unambiguously assigned to an SIG are represented by ×. The numbers on the *x*‐axis name the specific waterbodies of the study region in southern Bavaria, Germany: 1) Abtsdorfer See, 2) Altwasser Osterbuchberg, 3) Almfischerweiher, 4) Alz at Trostberg, 5) Alz at Altenmarkt, 6) Ammersee, 7) Baggerweiher Übersee, 8) Chiemsee deepest section, 9) Chiemsee at Fraueninsel, 10) Chiemsee at Chieming, 11) Chiemsee at Felden, 12) Chiemsee at Prien, 13) Chiemsee at Seebruck, 14) Chiemsee at Übersee, 15) Eschenauer See, 16) Fischzucht Eulenau, 17) Fischzucht Jäckle, 18) Fischzucht Kreißnig, 19) Fischzucht Weiß, 20) Hartsee, 21) Höglinger Baggersee, 22) Inn Rosenheim before Mangfall entry, 23) Inn Rosenheim after Mangfall entry, 24) Inn at Griesstätt, 25) Isar at Bad Tölz, 26) Isar after Loisach entry, 27) Klostersee, 28) Kratzsee, 29) Langbürgener See, 30) Lech at Landsberg, 31) Loisach at Wolfratshausen, 32) Mangfall at Bruckmühl, 33) Obinger See, 34) Pelhamer See, 35) Pilsensee, 36) Prien at Prien, 37) Salzach, 38) Schillinger See, 39) Seehamer See, 40) Simssee, 41) Starnberger See, 42) Tachinger See, 43) Tinninger See, 44) Tiroler Ache at Staudach, 45) Tiroler Ache at Unterwössen/Marquartstein, 46) Tiroler Ache at Übersee, 47) Traun at Traunstein, 48) Tüttensee, 49) Überseer Bach, 50) Waginger See, 51) Weißach, 52) Weitsee, 53) Wörthsee.

**Figure 5:**
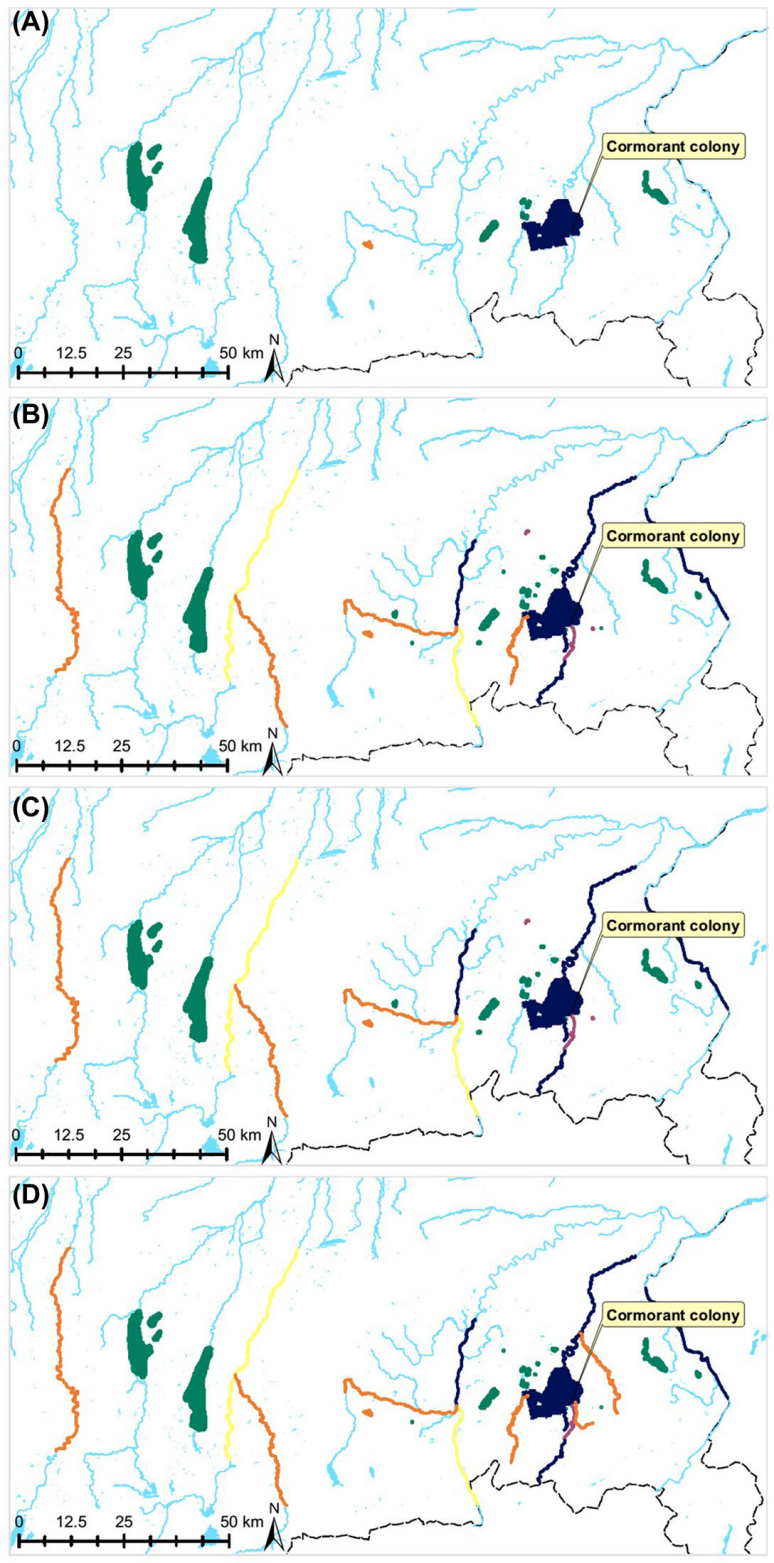
Waterbodies of the study region in southern Bavaria, Germany, 2012–2014, grouped in different habitat clusters (HC) based on their microchemistry for the fish taxa whitefish (A), cyprinids (B), perch (C), and salmonids (D). Colors indicate the taxon‐specific waterbodies in each habitat cluster (HC 1 = dark blue, HC 2 = green, HC 3 = orange, HC 4 = yellow, HC 5 = purple, HC 6 = brown; HC 6 not noticeable because of the small size of the respective single waterbody).

As a basis for assigning fish otoliths to habitat clusters with similar chemical characteristics, in a first step we identified 4 clearly distinguishable habitat strontium isotope groups (SIGs) based on the ^87^Sr/^86^Sr isotope ratios (Figure 4). The ^87^Sr/^86^Sr values in these SIGs including expanded uncertainties ranged from 0.7076–0.7081 (SIG 1, *n* = 4 waterbodies), 0.7082–0.7088 (SIG 2, *n* = 15 waterbodies), 0.7089–0.7093 (SIG 3, *n* = 2 waterbodies), and 0.7093–0.7102 (SIG 4, *n* = 3 waterbodies). Fish from these SIGs are expected to be well discriminated by their ^87^Sr/^86^Sr isotope ratios. We could not assign 29 bodies (55% of the waterbodies) to an SIG because of overlapping uncertainties. In a next step, we identified up to 6 taxon‐specific habitat clusters based on the ^87^Sr/^86^Sr isotope and Sr/Ca ratios in water samples (Figure 5; Figures S2–S5, available online in Supporting Information). For building these taxon‐specific clusters, we considered only those habitats where the respective fish taxa were expected to occur. Based on the microchemical signature of the water, we could identify for each taxon ≥1 habitat cluster that included Chiemsee (Figure 5).

Of 30 whitefish of known origin fed to captive cormorants, we found and analyzed 3 intact otoliths in the birds' pellets. We could allocate all of them to the habitat cluster (HC) including Chiemsee (Figure S2).

To determine the accuracy of the assignment per taxon, we assigned otoliths of the respective taxon with known origin (data derived from Zitek et al. [2021]) to the taxon‐specific habitat clusters. The accuracy of this assignment ranged between 95% for whitefish and 56% for salmonids (Table 1).

**Table 1:** Percentage of otoliths of known origin (data obtained from Zitek et al. [2021]) correctly assigned to the respective habitat cluster (HC) in the study region of Chiemsee in Germany, 2012–2014, and share of otoliths collected from cormorant pellets assigned to the species‐specific HC. The values in brackets are the percentage for correctly assigned otoliths of known origin when excluding otoliths of individuals showing a deviant otolith‐microchemistry because of stocking or migration. For each taxon, HC 1 represents the HC including Chiemsee.

	Otoliths of known origin	Otoliths from cormorants pellets					
	Correctly assigned to respective HC (%)	Assigned to HC 1 (%)	Assigned to HC 2 (%)	Assigned to HC 3 (%)	Assigned to HC 4 (%)	Assigned to HC 5 (%)	Assigned to HC 6 (%)
Whitefish	95	90.5	9.5	0.0			
Perch	71 [79]	58.4	33.3	4.9	2.2	1.1	
Cyprinids	78 [83]	54.4	34.0	2.9	1.9	6.8	
Salmonids	56 [75]	26.4	35.8	24.5	0.0	9.4	3.8

To analyze the origin of the cormorant prey out of 597 field‐collected cormorant pellets, we analyzed and assigned 628 otoliths from the taxa cyprinids, salmonids, whitefish, perch, burbot, eel, pike, pikeperch, or ruffe to the respective taxon‐specific habitat clusters (Table 1). Out of the field‐collected prey otoliths, we assigned 64% (*n* = 401) to a habitat cluster including Chiemsee and 36% (*n* = 226) to a habitat cluster excluding Chiemsee. We assigned otoliths out of pellets containing lake species only to the habitat cluster including Chiemsee significantly more often (68%) than otoliths out of pellets including riverine species (43%; χ^2^ = 24.847, *P* < 0.001; Figure 3B, C).

For otoliths of whitefish (*n* = 189), we assigned 90.5% to the habitat cluster including Chiemsee, while for otoliths of perch (*n* = 267), cyprinids (*n* = 103) and salmonids (*n* = 53) we assigned 58.4%, 54.4% and 26.4% to the habitat cluster including lake Chiemsee, respectively (Figures S2–S5; Table 1).

We assigned all of the analyzed otoliths of burbot and eel to the habitat cluster including Chiemsee, while the otoliths of pikeperch were assigned to a habitat cluster excluding Chiemsee. Out of the 6 analyzed otoliths of pike, we assigned 3 to the cluster including Chiemsee.

All pellets containing remains of ruffe (*n* = 14) were of notable interest because this fish species does not occur in Chiemsee or any other waterbody in a 25‐km range around the studied cormorant colony. In 11 of these pellets, we assigned the measured otolith to a habitat cluster including lake Ammersee or the rivers Inn or Lech, which are known habitats of ruffe. For the remaining 3 pellets containing ruffe remains, we assigned the measured otoliths to a habitat cluster consisting of small lakes and running waters around Chiemsee, which are not habitats for ruffe species.

From 30 pellets containing riverine species, we analyzed 2 otoliths of different prey species per pellet to test if both fish species were taken from the same waterbody. Of these paired otoliths, 37% (*n* = 11) had a common habitat cluster of waterbodies, while 63% (*n* = 19) were not assigned to a common cluster of waterbodies. In 83% (*n* = 25) of these pellets, we assigned ≥1 of the otoliths to the habitat cluster including Chiemsee.

## DISCUSSION

The present findings support our assumption that the Sr elemental and Sr isotopic composition of otoliths persist after digestion by seabirds and show that otoliths of unknown origin can be provenanced at an ecologically relevant scale even within a small‐scale region with limited geological variability. The feeding experiment showed that although exposed to digestive juices of seabirds, the microchemistry and isotope ratio of otoliths was not altered significantly so that all of the recovered otoliths of whitefish were provenanced correctly. Our findings support Phelps et al. (2009) and Kemp et al. (2011), who also reported no changes in microchemistry of otoliths digested by fish (largemouth bass [*Micropterus salmoides*]) and Australian fur seals (*Arctocephalus pusillus doriferus*).

We did not expect an unambiguous assignment of fish to the single sampled habitats given the limited variability of the ^87^Sr/^86^Sr isotope and Sr/Ca ratios in the different waterbodies. Depending on the taxon, however, we could use 3 to 6 habitat clusters for the classification. Although intermediate habitats might introduce some ambiguity to the results of the assignment per taxon, the accuracy of the assignment tested via otoliths of known origin ranged between 95% and 56%. Besides, Zitek et al. (2021) measured mismatches between ^87^Sr/^86^Sr isotope values of otoliths and their waterbodies of origin and identified respective fish as most likely to be stocked or migrated. This suggests that the differences in assignment accuracy do not directly depend on the taxon but on the potential to change habitat. In our study region, whitefish are typical lake species with a very low potential to migrate out of the inhabited lake and are predominantly stocked as young‐of‐the‐year, if at all. Perch and cyprinids such as roach and rudd occur in most of the stagnant waters of the study region, but also inhabit slowly running or dammed sections of rivers (Kottelat and Freyhof 2007). These species undertake short spawning migrations only (Kottelat and Freyhof 2007) and are rarely stocked. In contrast, salmonids such as trout and grayling have a higher potential to switch habitats as they undertake spawning migrations (Kottelat and Freyhof 2007) and are highly stocked because of their value for sport fishing. Therefore, trout and grayling have an increased probability to originate from another waterbody or another river section than they have been caught. The significant difference regarding provenancing accuracy between whitefish and other salmonids indicates that fish taxa with a high potential of habitat switching can be provenanced with lower accuracy than fish taxa that are not migratory and are rarely stocked. This is important to consider when it comes to the interpretation of results and the selection of the fish species to be examined in future studies.

The prey remains from pellets collected at a cormorant colony are often assigned to the waterbody where the colony is located (Rudstam et al. 2004, Stewart et al. 2005, Diana et al. 2006, Gwiazda and Amirowicz 2010, Emmrich and Düttmann 2011). For the cormorant colony at Chiemsee, this assumption is supported because the majority of the pellets (78%) contained solely lacustrine species. Based on the morphological identification of the prey, it could be assumed that in only 4% of the pellets the prey stemmed at least partially from flowing waters and consequently from waters other than Chiemsee. Microchemical and isotopic analysis show that in 32% of the pellets, prey fish came from waterbodies distinct from Chiemsee, even if only lake species were detected in the pellets. Also, 43% of the pellets including riverine species were assigned to the habitat cluster including Chiemsee. This may be because, depending on the taxon, the habitat cluster including Chiemsee also includes running waters and the analyzed otoliths stem from fish originating from these rivers. Moreover, some taxa occur in rivers and stagnant waters; salmonids such as the Alpine char (*Salvelinus umbla*) or the lacustrine form of brown trout (*Salmo trutta lacustris*) inhabit large alpine and pre‐alpine lakes (Kottelat and Freyhof 2007) and the rainbow trout is also stocked in various stagnant waters and lakes (Kottelat and Freyhof 2007). The otoliths of these trout species are not always clearly distinguishable from each other, especially when the otolith shape is altered by digestion. Therefore, based on the morphological species identification, for salmonids it cannot be completely ruled out that they originate from stagnant water. Alpine char and the lacustrine form of brown trout inhabit cold and deep sections of lakes (Kottelat and Freyhof 2007), and are thus not easily available for cormorants. In the study region, density and availability of trout is much higher in running waters and so is the probability that trout prey remains stem from fish dwelling in rivers. We assigned the majority (73%) of the analyzed otoliths from salmonids to a habitat cluster not containing Chiemsee.

Cyprinids include typical lake species, riverine species, and ubiquitous species. Among these fish, the lapillus is the most common type of otolith that was detected in pellets, but it cannot be identified to species level. Therefore, when analyzing cyprinid otoliths, it cannot be determined whether the otolith is from a typical riverine species or a typical lake species. This affects the approach for cyprinids because the number of potential habitats cannot be reduced and consequently each habitat cluster consists of a high number of habitats. Nevertheless, we assigned more than half of the cyprinid otoliths examined (53.9%) to the habitat cluster including Chiemsee.

Perch, a ubiquitous species, can be assumed to be distributed in all waterbodies of the study area. Therefore, the fact that the predominant (58%) origin of the prey was the habitat cluster including Chiemsee and large surrounding rivers is just as plausible as the remaining 42% being from the habitat cluster consisting of surrounding lakes and rivers farther away from Chiemsee. In contrast, for whitefish more than 90% of the otoliths examined could be clearly assigned to the Chiemsee habitat cluster. Whitefish are typical lake species, which are not present in rivers or small lakes and ponds of the study region. Therefore, the number of potential habitats is reduced and Chiemsee forms a discrete habitat cluster. We conclude that the species composition of a pellet provides information to which habitat the prey could have come from, but the exact habitat or even the waterbody of origin cannot be identified from the fish species composition.

Assuming that cormorants meet their daily food requirements in the waters where the colony is located (Rudstam et al. 2004, Stewart et al. 2005, Diana et al. 2006, Gwiazda and Amirowicz 2010, Emmrich and Düttmann 2011), otoliths from the same pellet should originate from the same cluster of waterbodies. Our results showed that in 63% of the cases where 2 otoliths from the same pellet were analyzed, the otoliths were assigned to different habitat clusters. This indicates that cormorants use different foraging grounds during 1 day because a pellet is considered to contain the indigestible prey remains of the previous day's consumed fish (Zijlstra and van Eerden 1995). Our findings confirm the results of previous researchers, which suggested that cormorants forage in waterbodies 11–60 km away from their breeding colonies (Warke et al. 1994, Grémillet et al. 1995, Engström 2001). Bugajski et al. (2013) observed that double‐crested cormorants switch foraging grounds on a daily and seasonal basis. They hypothesized that cormorants switch foraging grounds when the preferred type or size of prey is more easily available there. In waterbodies with strong anthropogenic activity regarding traffic, recreational use, or hazing, birds might temporarily also move to less‐disturbed waterbodies (Velando and Munilla 2011, Chatwin et al. 2013, Hentati‐Sundberg et al. 2021). In addition, young birds, non‐breeders, and roosting birds are reported to roam a region and visit several waterbodies (King 1996, Rutschke 1998, Leib 2009). Thus, the pellets of these roaming birds rather reflect the foraging grounds along a migration route than the waterbodies the birds switch between.

Provenancing fish otoliths offers various applications for addressing questions on the ecology and management of piscivores and their fish prey. For example, the waterbody from which the fish prey is taken plays a key role regarding the assessment of the impact piscivores exert on fish populations. Using cormorants as an example, Bugajski et al. (2013) state that it can be invalid to assume that all fish is taken from the waterbody where the bird colony is located. Using this assumption, there is the risk that the fish biomass removal tends to be overestimated for waterbodies with a cormorant colony nearby and that the predation pressure on fish in more distant waterbodies is underestimated. To predict the effects of piscivores on fish populations and fisheries, Bugajski et al. (2013) propose to collect the type, size, age of the prey, and the site‐specific fish biomass removal. By the use of microchemical provenancing in combination with information on fish populations and fish biomass, site‐specific predation measures can be obtained to develop management plans for maintaining fish populations or to regulate piscivores.

Our study provides conclusive evidence that microchemical provenancing of fish prey remains works on comparable small geographical scales so that fish from distinct clusters of waterbodies can be distinguished from others. This approach requires that fish hard parts, preferably otoliths, are obtainable from dietary samples of the examined piscivore. The spatial resolution of the assignment strongly depends on the differences in Sr isotopic and elemental composition of water in the potential habitats of fish and to what extend the waterbodies are connected. The resolution is likely to be highest in a study region consisting of isolated waterbodies with a significantly different geological background or catchment. Our findings also suggest that the assignment accuracy is higher for otoliths of fish species that rarely migrate or are not stocked.

## MANAGEMENT IMPLICATIONS

We show that microchemical provenancing of undigested fish otoliths provides a non‐invasive and small‐scale approach to identifying the prey origin. By combining prey species identification with provenancing of fish otoliths, it can be assessed on which prey species and habitats the birds rely on and to what extent fisheries and cormorants compete for the same fish resources. This allows managers to identify if the protection of certain fish populations or fish habitats from the birds is necessary and, if so, to develop site‐specific management plans. In turn, by provenancing cormorant prey the effect of management measures on the birds' prey choice can be investigated. For example, after hazing actions at certain foraging grounds, managers can identify which prey species and which foraging grounds the birds' switch to and can estimate the effect of the management measures on different fish habitats and populations.

For the management of the cormorants in the study region, it is important to consider the current findings, indicating that 36% of the fish prey is taken from waterbodies other than Chiemsee. This has to be considered when calculating the fish biomass removal by cormorants out of Chiemsee and estimating the maximum number of cormorants the fish productivity of the lake can support. Our findings also suggest that rather than reducing the number of birds in a central roosting or breeding colony, certain fish species or populations in specific waterbodies can be protected from predation by scaring off cormorants from the respective waterbodies and foraging sites around a bird colony. For example, to protect riverine fish populations from bird predation in our study region, measures have to be taken at the respective river and not at the breeding and roosting site.

## CONFLICTS OF INTEREST

The authors declare no conflicts of interest.

## ETHICS STATEMENT

In our study we followed the guidelines for the ethical treatment of birds by the Ornithological Council (Fair et al. 2010) especially regarding the impact of investigator presence and captive management. We did not catch or harm any of the wild birds in the cormorant colony. The investigator presence while collecting pellets underneath the nests was kept to the necessary minimum. The responsible zookeeper supervised the feeding trial with the captive cormorants in the zoological botanical garden Wilhelma Stuttgart and the respective aviaries were only entered for feeding, collecting the dietary samples, and cleaning.

*Associate Editor: Bill Block*.

## Supporting information

Additional supporting information may be found in the online version of the article at the publisher's website.

Supporting information.

## Data Availability

The data that support the findings of this study are available on request from the corresponding author.
