# Supplementary material for: Microchemical provenancing of prey remains in cormorant pellets reveals the use of diverse foraging grounds
Source: J Wildl Manage. 2022 May 8;86(6):e22248. doi: 10.1002/jwmg.22248 (PMC9544984; doi:10.1002/jwmg.22248)
Supplement: Supplementary file 1 — Supporting information. [file JWMG-86-0-s001.docx]

**SUPPORTING INFORMATION**

**25 Apr 2022**

**Oehm et al. 2022. Microchemical provenancing of prey remains in cormorant pellets reveals the use of diverse foraging grounds. The Journal of Wildlife Management**

Table S1: Numbered water sampling sites in a 50 km radius around Chiemsee and Ammersee in Germany, 2012-2014, including name of the site, category of water body, sampling date as well as latitude and longitude. Sampling was carried out in a period of two-and-a-half years beginning with the largest water bodies around Chiemsee and Ammersee in a pilot study in 2011 and then extended to all waterbodies around Chiemsee, including springwater from local hatcheries.

| **Habitat No.** | **Site name** | **Category** | **sampling date** | **Latitude** | **Longitude** |
| --- | --- | --- | --- | --- | --- |
| 1 | Abstdorfer See | lake | 21.11.2012 | 47.9145880000 | 12.9058270000 |
| 2 | Altwasser Osterbuchberg | oxbow | 21.11.2012 | 47.8095440000 | 12.5030530000 |
| 3 | Almfischerweiher | lake | 21.11.2012 | 47.8157842900 | 12.4993997800 |
| 4 | Alz at Trostberg | river | 10.03.2011 | 48.0306014000 | 12.5621918000 |
| 5 | Alz at Altenmarkt | river | 21.11.2012 | 48.0037195500 | 12.5334117900 |
| 6 | Ammersee | lake | 10.03.2011 | 47.9489825300 | 11.1110728100 |
| 7 | Baggerweiher Übersee | lake | 21.11.2012 | 47.8479243600 | 12.4867716100 |
| 8 | Chiemsee deepest section | lake | 03.09.2013 | 47.8885911700 | 12.4585163800 |
| 9 | Chiemsee at Fraueninsel | lake | 03.09.2013 | 47.8830771400 | 12.4174240100 |
| 10 | Chiemsee at Chieming | lake | 03.09.2013 | 47.8858769500 | 12.5054266200 |
| 11 | Chiemsee at Felden | lake | 03.09.2013 | 47.8411968700 | 12.3868367700 |
| 12 | Chiemsee at Prien | lake | 03.09.2013 | 47.8666129800 | 12.3708546600 |
| 13 | Chiemsee at Seebruck | lake | 03.09.2013 | 47.9214206600 | 12.4742685700 |
| 14 | Chiemsee at Übersee | lake | 03.09.2013 | 47.8480869700 | 12.4689058300 |
| 15 | Eschenauer See | lake | 08.02.2013 | 47.9466061800 | 12.3994098800 |
| 16 | Fischzucht Eulenau | hatchery | 21.11.2012 | 47.8297440900 | 11.9947795400 |
| 17 | Fischzucht Jäckle | hatchery | 21.11.2012 | 47.9231391100 | 12.3877403400 |
| 18 | Fischzucht Kreißnig | hatchery | 21.11.2012 | 47.9769625800 | 12.2922049200 |
| 19 | Fischzucht Weiß | hatchery | 21.11.2012 | 47.8493551500 | 12.5964877000 |
| 20 | Hartsee | lake | 21.11.2012 | 47.9294198400 | 12.3739049200 |
| 21 | Höglinger Baggerseen | lake | 08.02.2013 | 47.8905952000 | 11.9419209100 |
| 22 | Inn Rosenheim before Mangfall entry | river | 08.02.2013 | 47.8586973700 | 12.1370196800 |
| 23 | Inn Rosenheim after Mangfall entry | river | 08.02.2013 | 47.8631735500 | 12.1349020800 |
| 24 | Inn at Griesstätt | river | 08.02.2013 | 47.9951873600 | 12.1634072500 |
| 25 | Isar at Bad Tölz | river | 10.03.2011 | 47.7613625200 | 11.5563510200 |
| 26 | Isar after Loisach entry | river | 10.03.2011 | 47.9735086500 | 11.4752687600 |
| 27 | Klostersee | lake | 08.02.2013 | 47.9724316600 | 12.4577475100 |
| 28 | Kratzsee | lake | 21.11.2012 | 48.0570270000 | 12.3643960000 |
| 29 | Langbürgener See | lake | 21.11.2012 | 47.8950297300 | 12.3607583300 |
| 30 | Lech at Landsberg | river | 10.03.2011 | 48.0497797000 | 10.8718378700 |
| 31 | Loisach at Wolfratshausen | river | 10.03.2011 | 47.9123651800 | 11.4189299700 |
| 32 | Mangfall at Bruckmühl | river | 08.02.2013 | 47.8803080900 | 11.9113159800 |
| 33 | Obingersee | lake | 08.02.2013 | 48.0014477200 | 12.4154272800 |
| 34 | Pelhamer See | lake | 08.02.2013 | 47.9334553900 | 12.3436635200 |
| 35 | Pilsensee | lake | 10.03.2011 | 48.0206548500 | 11.1926332500 |
| 36 | Prien at Prien | river | 03.09.2013 | 47.8537883400 | 12.3350616700 |
| 37 | Salzach | river | 10.03.2011 | 47.9396817200 | 12.9394310100 |
| 38 | Schillinger See | lake | 08.02.2013 | 48.0622236400 | 12.3762241900 |
| 39 | Seehamer See | lake | 08.02.2013 | 47.8473751800 | 11.8646209000 |
| 40 | Simssee | lake | 10.03.2011 | 47.8576657200 | 12.2264503100 |
| 41 | Starnberger See | lake | 10.03.2011 | 47.9656072000 | 11.3222831900 |
| 42 | Tachinger See | lake | 10.03.2011 | 47.9885158600 | 12.7450347200 |
| 43 | Tinninger See | lake | 08.02.2013 | 47.8250090700 | 12.2102280600 |
| 44 | Tiroler Ache at Staudach | river | 10.03.2011 | 47.7801675700 | 12.4778509200 |
| 45 | Tiroler Ache at Unterwössen/Marquartstein | river | 21.11.2012 | 47.7383273700 | 12.4450855500 |
| 46 | Tiroler Ache at Übersee | river | 21.11.2012 | 47.8206262600 | 12.5050754200 |
| 47 | Traun at Traunstein | river | 10.03.2011 | 47.8653401900 | 12.6515673700 |
| 48 | Tüttensee | lake | 21.11.2012 | 47.8464973400 | 12.5661141200 |
| 49 | Überseer Bach | river | 21.11.2012 | 47.8375607200 | 12.4810522200 |
| 50 | Waginger See | lake | 10.03.2011 | 47.9226382100 | 12.7922100000 |
| 51 | Weißach | river | 21.11.2012 | 47.8271365700 | 12.5136386200 |
| 52 | Weitsee | lake | 08.02.2013 | 48.0586150600 | 12.3686030300 |
| 53 | Wörthsee | lake | 10.03.2011 | 48.0553237400 | 11.1850244100 |

**
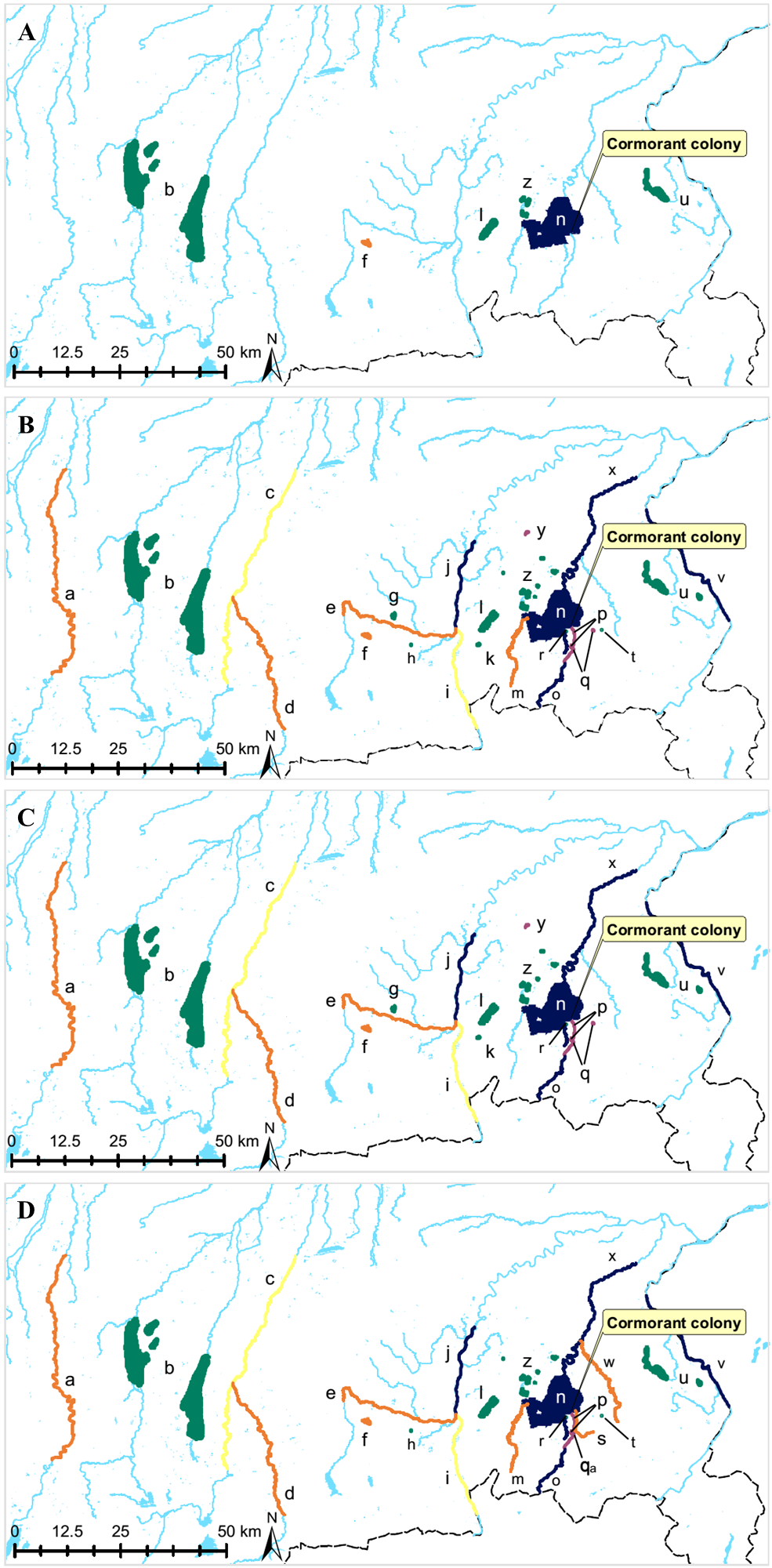
**

Figure S1. Waterbodies of the study region grouped in different habitat clusters (HC) based on their microchemistry for the fish taxa whitefish (A), cyprinids (B), perch (C) and salmonids (D), respectively. Letters name the water bodies per habitat cluster: u) Abtsdorfer See, q_a_) Altwasser Osterbuchberg, p) Almfischerweiher, x) Alz at Trostberg, x) Alz at Altenmarkt, b) Ammersee, r) Baggerweiher Übersee, n) Chiemsee deepest section, n) Chiemsee at Fraueninsel, n) Chiemsee at Chieming, n) Chiemsee at Felden, n) Chiemsee at Prien, n) Chiemsee at Seebruck, n) Chiemsee at Übersee, z) Eschenauer See, h) Fischzucht Eulenau, z) Fischzucht Jäckle, z) Fischzucht Kreißnig, t) Fischzucht Weiß, z) Hartsee, g) Höglinger Baggersee, i) Inn Rosenheim before Mangfall entry, j) Inn Rosenheim after Mangfall entry, j) Inn at Griesstätt, d) Isar at Bad Tölz, c) Isar after Loisach entry, z) Klostersee, y) Kratzsee, z) Langbürgener See, a) Lech at Landsberg, c) Loisach at Wolfratshausen, e) Mangfall at Bruckmühl, z) Obinger See, z) Pelhamer See, b) Pilsensee, m) Prien at Prien, v) Salzach, y) Schillinger See, f) Seehamer See, l) Simssee, b) Starnberger See, u) Tachinger See, k) Tinninger See, o) Tiroler Ache at Staudach, o) Tiroler Ache at Unterwössen/Marquartstein, p) Tiroler Ache at Übersee, w) Traun at Traunstein, q) Tüttensee, o) Überseer Bach, u) Waginger See, s) Weißach, y) Weitsee, b) Wörthsee. Please note that for a labelled display of the habitats it was necessary to summarized some habitats and introduce lower case letters as labels to avoid overcrowding the figure.


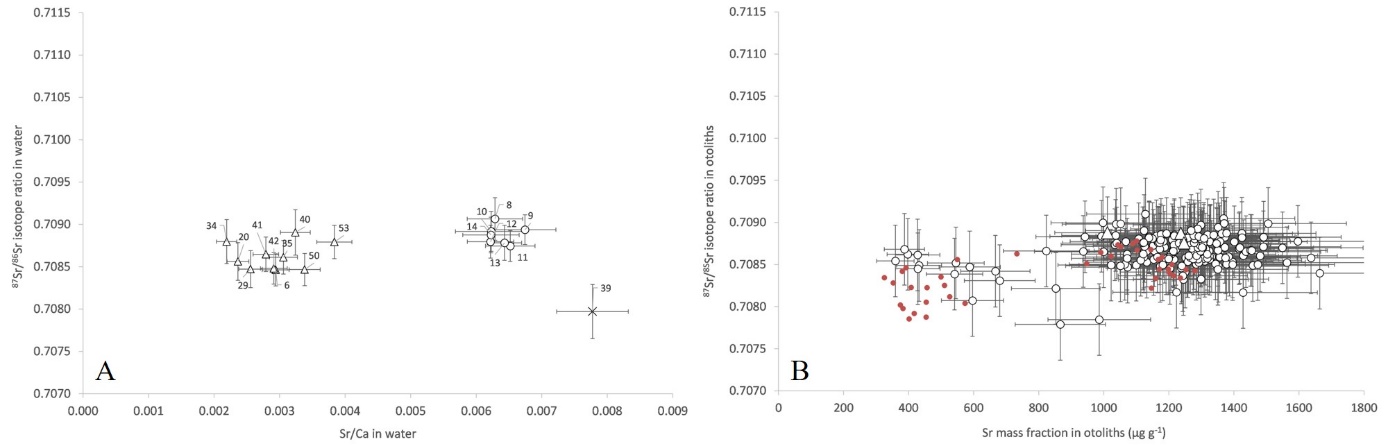


Figure S2. Habitat clusters (HC; n=3) for whitefish (*Coregonus spp.*) in the study region in Germany, 2012-2014, based on ^87^Sr/^86^Sr isotope and Sr/Ca ratios of water samples (A) and the plotted otolith data (B). Numbers in A name the specific water bodies: 1) Abtsdorfer See, 2) Altwasser Osterbuchberg, 3) Almfischerweiher, 4) Alz at Trostberg, 5) Alz at Altenmarkt, 6) Ammersee, 7) Baggerweiher Übersee, 8) Chiemsee deepest section, 9) Chiemsee at Fraueninsel, 10) Chiemsee at Chieming, 11) Chiemsee at Felden, 12) Chiemsee at Prien, 13) Chiemsee at Seebruck, 14) Chiemsee at Übersee, 15) Eschenauer See, 16) Fischzucht Eulenau, 17) Fischzucht Jäckle, 18) Fischzucht Kreißnig, 19) Fischzucht Weiß, 20) Hartsee, 21) Höglinger Baggersee, 22) Inn Rosenheim before Mangfall entry, 23) Inn Rosenheim after Mangfall entry, 24) Inn at Griesstätt, 25) Isar at Bad Tölz, 26) Isar after Loisach entry, 27) Klostersee, 28) Kratzsee, 29) Langbürgener See, 30) Lech at Landsberg, 31) Loisach at Wolfratshausen, 32) Mangfall at Bruckmühl, 33) Obinger See, 34) Pelhamer See, 35) Pilsensee, 36) Prien at Prien, 37) Salzach, 38) Schillinger See, 39) Seehamer See, 40) Simssee, 41) Starnberger See, 42) Tachinger See, 43) Tinninger See, 44) Tiroler Ache at Staudach, 45) Tiroler Ache at Unterwössen/Marquartstein, 46) Tiroler Ache at Übersee, 47) Traun at Traunstein, 48) Tüttensee, 49) Überseer Bach, 50) Waginger See, 51) Weißach, 52) Weitsee, 53) Wörthsee. B is a bi-plot of whitefish otoliths of known origin (filled/red dots) and of whitefish otoliths from cormorant pellets (unfilled dots) with three of them from the feeding experiment (triangle).


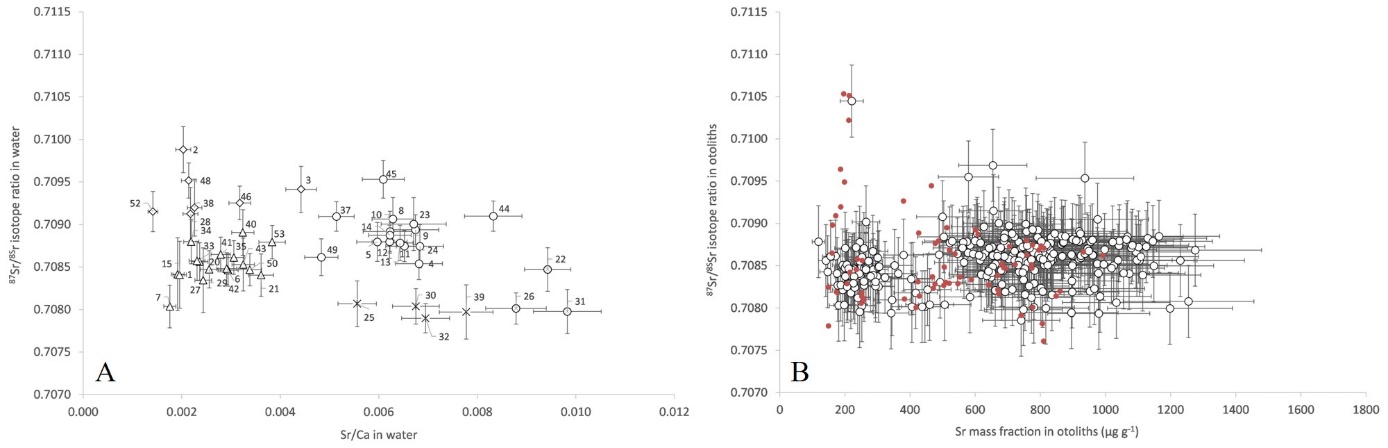


Figure S3. Habitat clusters (HC; n=5) for perch (*Perca fluviatilis*) in the study region in Germany, 2012-2014, based on ^87^Sr/^86^Sr isotope and Sr/Ca ratios of water samples (A) and the plotted otolith data (B). Numbers in A name the specific water bodies: Numbers in A name the specific water bodies: 1) Abtsdorfer See, 2) Altwasser Osterbuchberg, 3) Almfischerweiher, 4) Alz at Trostberg, 5) Alz at Altenmarkt, 6) Ammersee, 7) Baggerweiher Übersee, 8) Chiemsee deepest section, 9) Chiemsee at Fraueninsel, 10) Chiemsee at Chieming, 11) Chiemsee at Felden, 12) Chiemsee at Prien, 13) Chiemsee at Seebruck, 14) Chiemsee at Übersee, 15) Eschenauer See, 16) Fischzucht Eulenau, 17) Fischzucht Jäckle, 18) Fischzucht Kreißnig, 19) Fischzucht Weiß, 20) Hartsee, 21) Höglinger Baggersee, 22) Inn Rosenheim before Mangfall entry, 23) Inn Rosenheim after Mangfall entry, 24) Inn at Griesstätt, 25) Isar at Bad Tölz, 26) Isar after Loisach entry, 27) Klostersee, 28) Kratzsee, 29) Langbürgener See, 30) Lech at Landsberg, 31) Loisach at Wolfratshausen, 32) Mangfall at Bruckmühl, 33) Obinger See, 34) Pelhamer See, 35) Pilsensee, 36) Prien at Prien, 37) Salzach, 38) Schillinger See, 39) Seehamer See, 40) Simssee, 41) Starnberger See, 42) Tachinger See, 43) Tinninger See, 44) Tiroler Ache at Staudach, 45) Tiroler Ache at Unterwössen/Marquartstein, 46) Tiroler Ache at Übersee, 47) Traun at Traunstein, 48) Tüttensee, 49) Überseer Bach, 50) Waginger See, 51) Weißach, 52) Weitsee, 53) Wörthsee. B is a bi-plot of whitefish otoliths of known origin (filled/red dots) and of whitefish otoliths from cormorant pellets (unfilled dots),


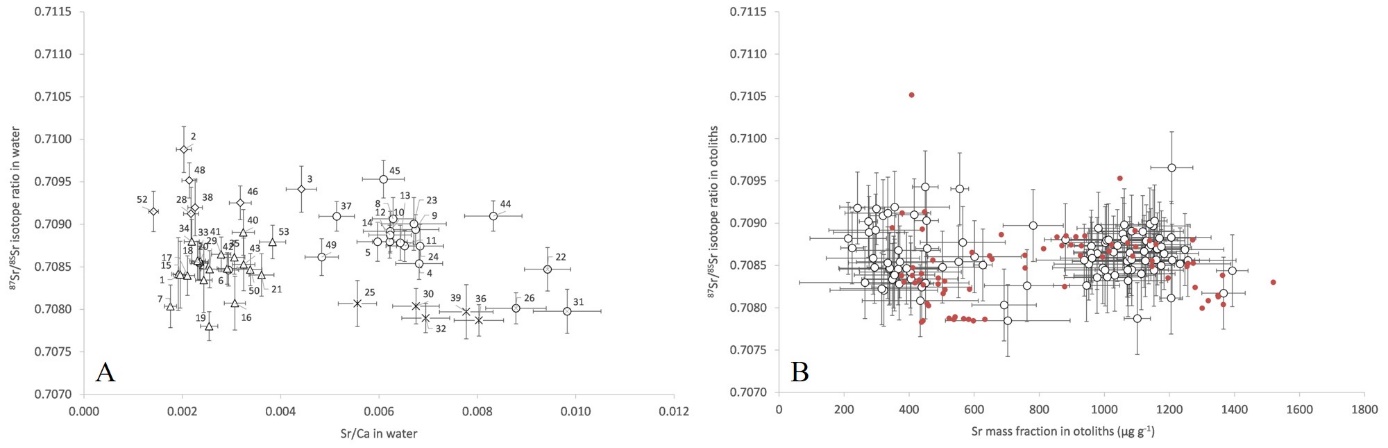


Figure S4: Habitat clusters (HC; n=5) for cyprinids in the study region in Germany, 2012-2014, based on ^87^Sr/^86^Sr isotope and Sr/Ca ratios of water samples (A) and the plotted otolith data (B). Numbers in A name the specific water bodies: 1) Abtsdorfer See, 2) Altwasser Osterbuchberg, 3) Almfischerweiher, 4) Alz at Trostberg, 5) Alz at Altenmarkt, 6) Ammersee, 7) Baggerweiher Übersee, 8) Chiemsee deepest section, 9) Chiemsee at Fraueninsel, 10) Chiemsee at Chieming, 11) Chiemsee at Felden, 12) Chiemsee at Prien, 13) Chiemsee at Seebruck, 14) Chiemsee at Übersee, 15) Eschenauer See, 16) Fischzucht Eulenau, 17) Fischzucht Jäckle, 18) Fischzucht Kreißnig, 19) Fischzucht Weiß, 20) Hartsee, 21) Höglinger Baggersee, 22) Inn Rosenheim before Mangfall entry, 23) Inn Rosenheim after Mangfall entry, 24) Inn at Griesstätt, 25) Isar at Bad Tölz, 26) Isar after Loisach entry, 27) Klostersee, 28) Kratzsee, 29) Langbürgener See, 30) Lech at Landsberg, 31) Loisach at Wolfratshausen, 32) Mangfall at Bruckmühl, 33) Obinger See, 34) Pelhamer See, 35) Pilsensee, 36) Prien at Prien, 37) Salzach, 38) Schillinger See, 39) Seehamer See, 40) Simssee, 41) Starnberger See, 42) Tachinger See, 43) Tinninger See, 44) Tiroler Ache at Staudach, 45) Tiroler Ache at Unterwössen/Marquartstein, 46) Tiroler Ache at Übersee, 47) Traun at Traunstein, 48) Tüttensee, 49) Überseer Bach, 50) Waginger See, 51) Weißach, 52) Weitsee, 53) Wörthsee. B is a bi-plot of cyprinid otoliths of known origin (filled/red dots) and of cyprinid otoliths from cormorant pellets (unfilled dots).


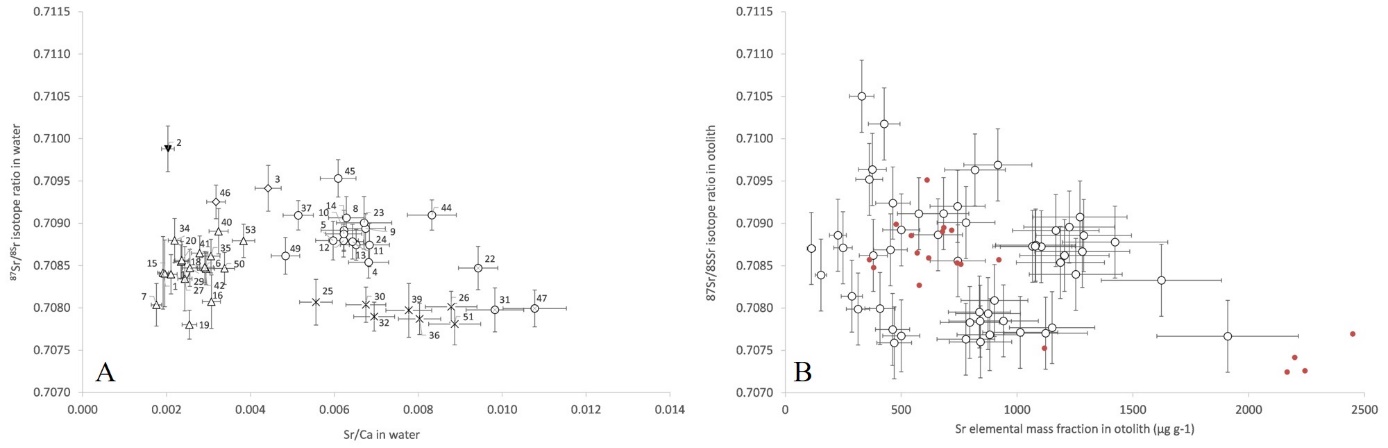


Figure S5. Habitat clusters (HC; n=6) for salmonids in the study region in Germany, 2012-2014, based on ^87^Sr/^86^Sr isotope and Sr/Ca ratios of water samples (A) and the plotted otolith data (B). Numbers in A name the specific water bodies: 1) Abtsdorfer See, 2) Altwasser Osterbuchberg, 3) Almfischerweiher, 4) Alz at Trostberg, 5) Alz at Altenmarkt, 6) Ammersee, 7) Baggerweiher Übersee, 8) Chiemsee deepest section, 9) Chiemsee at Fraueninsel, 10) Chiemsee at Chieming, 11) Chiemsee at Felden, 12) Chiemsee at Prien, 13) Chiemsee at Seebruck, 14) Chiemsee at Übersee, 15) Eschenauer See, 16) Fischzucht Eulenau, 17) Fischzucht Jäckle, 18) Fischzucht Kreißnig, 19) Fischzucht Weiß, 20) Hartsee, 21) Höglinger Baggersee, 22) Inn Rosenheim before Mangfall entry, 23) Inn Rosenheim after Mangfall entry, 24) Inn at Griesstätt, 25) Isar at Bad Tölz, 26) Isar after Loisach entry, 27) Klostersee, 28) Kratzsee, 29) Langbürgener See, 30) Lech at Landsberg, 31) Loisach at Wolfratshausen, 32) Mangfall at Bruckmühl, 33) Obinger See, 34) Pelhamer See, 35) Pilsensee, 36) Prien at Prien, 37) Salzach, 38) Schillinger See, 39) Seehamer See, 40) Simssee, 41) Starnberger See, 42) Tachinger See, 43) Tinninger See, 44) Tiroler Ache at Staudach, 45) Tiroler Ache at Unterwössen/Marquartstein, 46) Tiroler Ache at Übersee, 47) Traun at Traunstein, 48) Tüttensee, 49) Überseer Bach, 50) Waginger See, 51) Weißach, 52) Weitsee, 53) Wörthsee. B is a bi-plot of salmonid otoliths of known origin (filled/red dots) and of salmonid otoliths from cormorant pellets (unfilled dots).
